# Comparative Evaluation of Shear Bond Strength Between Monolithic Zirconia and Three Different Core Build-Up Materials

**DOI:** 10.3390/jfb16090355

**Published:** 2025-09-21

**Authors:** Ayben Şentürk, Bora Akat, Fehmi Gönüldaş, Onur Alp Halaçlı, Selin Kartal, Mehmet Ali Kılıçarslan

**Affiliations:** 1 Faculty of Dentistry, Prosthetic Dentistry, Ankara University, 06500 Ankara, Turkey; aybensenturk@ankara.edu.tr (A.Ş.); fgonuldas@ankara.edu.tr (F.G.); makilicarslan@ankara.edu.tr (M.A.K.); 2 Prosthetic Dentistry Program, Graduate School of Health Sciences, Ankara University, 06500 Ankara, Turkey; abdurrahmanonurhalacli@ankara.edu.tr (O.A.H.); slnkartal@ankara.du.tr (S.K.)

**Keywords:** zirconomer, core build-up, nanohybrid composite, monolithic zirconia, shear bond strength

## Abstract

This in vitro study aimed to evaluate and compare the shear bond strength (SBS) between monolithic zirconia (MZ) and different core build-up materials. Sixty cylindrical MZ specimens were fabricated and divided into three groups (*n* = 20) based on the type of core build-up material: nanohybrid composite resin (NHCR), glass ionomer cement (GIC), and zirconia-reinforced glass ionomer cement (zirconomer). All specimens were subjected to airborne-particle abrasion with aluminum oxide and bonded using a self-adhesive dual-cure resin cement. After 24 h of storage in distilled water at 37 °C, SBS testing was performed using a universal testing machine. Failure modes were examined under a stereomicroscope and classified as adhesive, cohesive, or mixed. The NHCR group exhibited the highest SBS values (48.32 ± 12.49 MPa), followed by the Zirkonomer group (14.19 ± 3.66 MPa), and the GIC group (10.37 ± 4.21 MPa). The SBS of NHCR was significantly higher than that of both Zirconomer and GIC (*p* < 0.001). Although no significant difference was found between Zirconomer and GIC, Zirconomer demonstrated higher mean bond strength. Within the limitations of this study, NHCR showed the highest bond strength to monolithic zirconia. Zirconomer performed better than conventional GIC; however, further investigations involving different surface treatments and long-term clinical conditions are recommended to enhance its bonding efficacy.

## 1. Introduction

Despite the increasing awareness of the importance of oral and dental health, the demand for endodontic treatment remains consistently prevalent [1]. The long-term prognosis of endodontically treated teeth is influenced by several key factors, such as the quality of the coronal seal, the number of remaining cavity walls, the presence of a ferrule, post placement, and the application of indirect restorations [1,2]. After endodontic treatment, core restorations play a crucial role in restoring the natural anatomical form of the tooth and reinforcing its strength to withstand functional and parafunctional stresses [2].

Core build-up is defined as a restorative procedure designed to enable the reconstruction of a significant portion of the coronal structure in teeth with extensive loss of hard tissue [3,4]. It is typically indicated when more than 50% of the crown structure is missing [5]. Since stress distribution differs between intact and restored teeth, compressive, tensile, and shear forces may simultaneously act on both the restoration and the tooth–restoration interface. Therefore, the mechanical strength of core build-up materials is a critical determinant for material selection [6].

An ideal core build-up material should demonstrate adequate compressive and flexural strength, ensure biocompatibility with surrounding tissues, and establish a durable bond with tooth structure, pins, posts, and luting cements. For this purpose, a variety of materials have been employed, including cast metal cores, amalgam, composite resins, glass ionomer cements (GICs), porcelain, and compomers [7,8]. Among these, composite resins are widely preferred due to their superior mechanical properties [2,9].

GICs have been widely used in core restorations for many years due to their advantageous properties such as chemical adhesion to hard dental tissues, reduced postoperative sensitivity, and fluoride release [10]. To overcome the mechanical limitations of conventional GICs, Zirconomer was recently introduced through the incorporation of zirconia particles, thereby combining the adhesive bonding capacity and fluoride release capability of GICs with improved mechanical strength and occlusal resistance, making it a promising alternative in restorative dentistry [3,11]. The powder component of Zirconomer contains fluoroaluminosilicate glass, zirconium oxide, and pigments, whereas the liquid component consists primarily of polyacrylic acid and tartaric acid [7,12].

For teeth with extensive structural loss, indirect full or partial coverage restorations are recommended to provide cuspal protection following direct restorations. The bond strength between the core build-up and the crown is a decisive factor in the long-term success of all-ceramic restorations [13,14]. In high-strength all-ceramic materials such as zirconia crowns, bonding quality directly affects mechanical stability and marginal integrity, making both micromechanical retention and chemical interaction with resin cements essential for clinical success. In the literature, numerous studies have investigated the effects of various surface treatments (e.g., sandblasting, silica coating, plasma treatment) and adhesive resin systems applied before cementation to improve the bond strength of zirconia crowns [15,16]. However, while most studies primarily focused on the zirconia substrate, there is limited evidence on the bonding behavior of zirconia crowns to underlying core build-up materials, and comparative data on materials such as GIC, composite resins, or modified formulations remain scarce [2,3,7,8]. Understanding the influence of core material selection is therefore critical for the long-term success of restorations in endodontically treated teeth.

The aim of this in vitro study was to evaluate and compare the shear bond strength between monolithic zirconia and three core build-up materials: NHCR, GIC, and Zirconomer. In addition, the distribution of failure modes (adhesive, cohesive, and mixed) at the bonding interfaces was analyzed. The null hypothesis stated that there would be no statistically significant difference in shear bond strength among the tested core build-up materials.

## 2. Materials and Methods

In this experimental in vitro study, 60 monolithic zirconia specimens were bonded to three different core build-up materials using a self-adhesive dual-cure resin cement, and their shear bond strength was evaluated. According to a power analysis (α = 0.05, power = 0.8), 60 square-shaped zirconia specimens and 60 disc-shaped core build-up specimens were fabricated and randomly allocated to three groups (*n* = 20 per group).

Monolithic zirconia blocks (inCoris TZI C disc 22 A1, Sirona Dental Systems, Bensheim, Germany; Batch No. 2016290053-1055) were cut from CAD/CAM blocks and sectioned using a precision micro-cutting machine (Struers Secotom-60, Struers A/S, Ballerup, Denmark). During the cutting process, the shrinkage expected after sintering was considered, and the specimens were prepared in larger dimensions to achieve the final size of 10 × 10 × 2 mm. Accordingly, the zirconia blocks were cut to approximately 12.5 × 12.5 × 2.5 mm prior to sintering. After sintering, the zirconia blocks shrank by approximately 20% and reached the targeted final dimensions. Sintering was performed at 1510 °C for 8 h.

Afterwards, only one surface of each zirconia specimen was subjected to sandblasting. Sandblasting was performed using 30 µm aluminum oxide (Al_2_O_3_) particles (CoJet, 3M Deutschland GmbH, Neuss, Germany) at 2 bar pressure, from a 10 mm distance, for 15 s. Sandblasting was chosen as the sole surface treatment method because it is considered essential for achieving sufficient bond strength in high-strength ceramics [15] and is also a common and clinically relevant approach widely adopted for conditioning zirconia surfaces [16]. The specimens were then divided into three groups according to the core build-up material to be tested (*n* = 20).

The disc-shaped core build-up specimens (n = 20 for each core build-up material) were prepared using a mold with dimensions of 10 mm × 4 mm. The core build-up specimens were prepared to the same size as the zirconia discs to standardize the bonding area for shear bond strength testing and to simulate the clinical situation where zirconia restorations fully cover the core surface. Three different core build-up materials, GIC (Ketac Molar, 3M ESPE, Seefeld, Germany, Batch No. 1114316), NHCR (RubyCompNano, Inci Dental, Istanbul, Turkey, Batch No. 5068), and Zirconomer (zirconia-reinforced glass ionomer, SHOFU, Kyoto, Japan, Batch No. 05220583), were placed into the mold using manual instruments.

Zirconomer specimens were prepared according to the manufacturer’s instructions by mixing at a 2:1 powder-to-liquid ratio on a glass slab with an agate spatula, then placed into the mold and left to self-cure at room temperature. Similarly, the Ketac Molar material used for the GIC group was mixed at a 2.5:1 powder-to-liquid ratio according to the manufacturer’s instructions, placed into the mold, and chemically self-cured. The NHCR (RubyCompNano) specimens were light-cured for 20 s according to the manufacturer’s instructions using a LED curing unit (Radii Plus, SDI, Bayswater, Australia; intensity: 1200–1500 mW/cm^2^; wavelength: 440–480 nm). The composition and manufacturer information of all materials are presented in Table 1.

**Figure 1:**
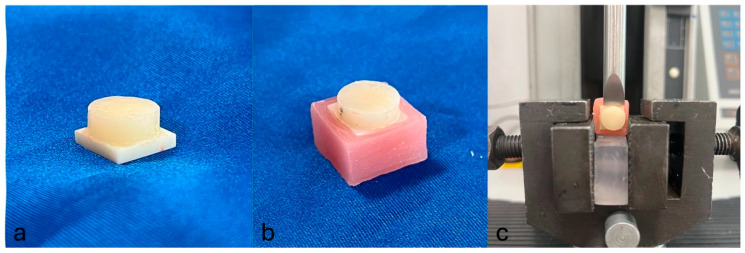
(**a**) Bonding of the core build-up material to the monolithic zirconia specimen, (**b**) specimen embedded in acrylic resin, and (**c**) evaluation of the specimen during shear bond strength testing using a universal testing machine.

Each specimen was examined for surface irregularities, and the final dimensions were confirmed using a precision caliper. The sandblasted surface of the monolithic zirconia specimens was covered with a self-etching and self-adhesive dual-cure resin cement containing the 10-MDP monomer (RubySE Cem, Inci Dental, Turkey, Batch No. RSC002), which is known to enhance chemical bonding to zirconia surfaces [8,16]. The core build-up materials were subsequently placed onto the zirconia specimens (Figure 1a). A constant load of 5 kg was applied to ensure uniform cement thickness, and excess cement was carefully removed. All specimens were light-cured for 20 s from four directions.

After cementation, the specimens were stored in distilled water at 37 °C for 24 h to simulate the moist and stable temperature conditions of the oral environment in vitro and to ensure complete polymerization and stabilization of the mechanical properties of both the core build-up materials and the resin cement. Subsequently, a mold was prepared using type C silicone (Zhermack, Italy) for embedding the specimens in acrylic resin (Ivoclar Vivadent, Liechtenstein). The cemented and water-stored specimens were embedded in acrylic blocks so that half of the thickness of the monolithic zirconia remained exposed above the acrylic surface (Figure 1b).

For the evaluation of bond strength, all specimens were subjected to a shear bond strength test. The test was performed using a universal testing machine (Lloyd Instruments, Model LRX, Bognor Regis, UK) operating at a crosshead speed of 1 mm/min until failure occurred at the bonding interface (Figure 1c). The maximum load (N) sustained by each specimen was recorded. This value was then divided by the bonding area (mm^2^) to calculate the shear bond strength in MPa. All statistical analyses were performed using the shear bond strength values expressed in MPa.

In addition, all specimens were examined for failure modes. Each specimen was evaluated and classified according to semi-quantitative criteria as one of three failure types: adhesive (score 0, >75% of the failure surface exposed), cohesive (score 2, >75% covered with residual core material or fracture within the core material), or mixed (score 1, neither criterion fully met). The evaluations were carried out using a stereomicroscope (LEICA MZ12, Leica Microsystems, Wetzlar, Germany) at 10× magnification. All specimens were evaluated and classified by a single experienced examiner (E1) who had previously participated in similar studies. Prior to the main evaluations, E1 underwent a calibration session using a set of representative stereomicroscopic images. A predefined scoring guide was used, including explicit decision rules and a visual aid to estimate the 75% threshold for class assignment (adhesive, cohesive, mixed). Standardized imaging conditions (magnification, illumination, and working distance) were established and maintained for all observations.

### Statistical Analysis

All statistical analyses were performed using IBM SPSS Statistics v26.0 (IBM Corp., Armonk, NY, USA). The normality of the data distribution was assessed using the Shapiro–Wilk test, while Levene’s test was used to evaluate the homogeneity of variances among the groups. Depending on the distribution and variance characteristics of the data, appropriate parametric or non-parametric tests were applied for group comparisons. The Kruskal–Wallis test was used to compare the shear bond strength values, and when a significant difference was found, pairwise comparisons were performed using the Bonferroni-corrected Dunn’s post hoc test. The distribution of failure modes was analyzed using Pearson’s Chi-square test. A significance threshold of *p* < 0.05 was adopted for all statistical evaluations.

## 3. Results

The descriptive statistics of the shear bond strength values for the tested core materials are summarized in Table 2. The highest mean bond strength was observed in the NHCR group (48.32 ± 12.49 MPa), followed by the zirconomer (14.19 ± 3.66 MPa) and GIC (10.37 ± 4.21 MPa) groups. The NHCR demonstrated significantly higher bond strength compared with both GIC and zirconomer groups (*p* < 0.001). Although the difference between the GIC and zirconomer groups was not statistically significant (*p* = 1.000), the zirconomer group exhibited higher bond strength values than GIC. These findings suggest that the NHCR exhibit superior bonding performance to zirconia surfaces compared to the other tested core materials.

**Figure 2:**
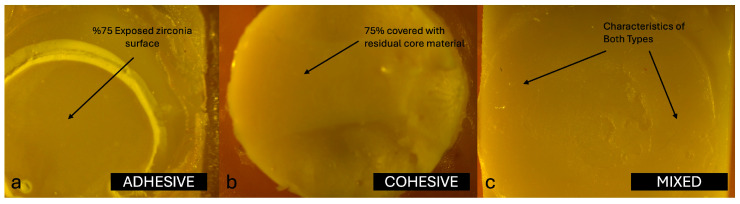
(**a**) Adhesive failure, (**b**) Cohesive failure, and (**c**) Mixed failure pattern of zirconomer group at 10× magnification of a stereomicroscope.

**Figure 3:**
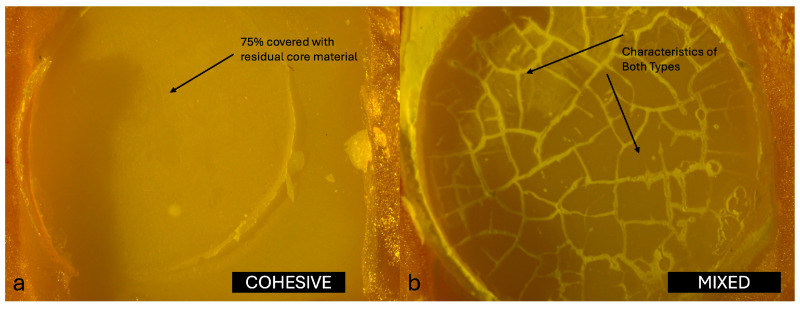
(**a**) Cohesive failure and (**b**) Mixed failure pattern of GIC group at 10× magnification of a stereomicroscope.

**Figure 4:**
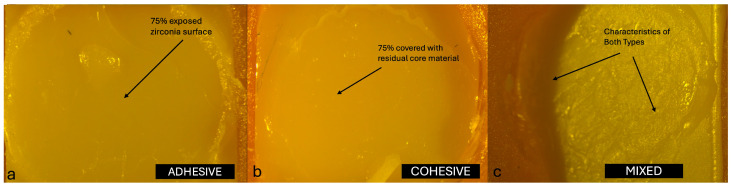
(**a**) Adhesive failure, (**b**) Cohesive failure, and (**c**) Mixed failure pattern of NHCR group at 10× magnification of a stereomicroscope.

The distribution of failure modes among the core materials is summarized as follows. In the Zirconomer group, failures were predominantly mixed (65%), followed by cohesive (30%) and adhesive (5%). The GIC group showed no adhesive failures, with 40% cohesive and 60% mixed failures. In the NHCR group, 15% of the failures were adhesive, 40% cohesive, and 45% mixed. Representative stereomicroscopic images of the typical failure patterns for each group are shown in Figure 2, Figure 3 and Figure 4. Statistical comparison of the distributions using the Chi-square test revealed no significant differences between groups (χ^2^ = 4.6283; df = 4; *p* = 0.327).

## 4. Discussion

In this in vitro study, the shear bond strength between monolithic zirconia and three core build-up materials (NHCR, GIC, and Zirconomer) was evaluated. NHCR showed the highest bond strength, while Zirconomer presented higher mean values than GIC, although the difference was not statistically significant. Accordingly, the null hypothesis stating that “there is no statistically significant difference in shear bond strength between monolithic zirconia and different core build-up materials” was rejected.

The superior properties of composite resins have been demonstrated in numerous studies [17,18,19,20,21], and their advantages over amalgam as core build-up materials have been frequently emphasized [17,18,21]. Agrawal and Mala [21] showed that nano-filled resin composites could serve as an effective alternative to amalgam core materials in terms of their mechanical properties. Tavakolizadeh et al. [13] evaluated the shear bond strength of zirconia bonded to various core build-up materials (non-precious gold alloy, zirconia ceramic, natural dentin, and composite resin) and found that composite resin achieved the highest values (11.58 ±1.74 MPa), confirming its status as the most favorable material. Among different composite types, NHCRs exhibit superior mechanical properties compared with microhybrid and microfill resins [3,19], which is consistent with our findings, where NHCR demonstrated the highest shear bond strength (48.32 ± 12.49 MPa).

Capp and Warren [18] reported that composite and acrylic resins can serve as acceptable alternatives to core build-up materials, whereas glass ionomer cements (GICs) are considered the weakest option in terms of mechanical performance. This observation underscores the inherent limitations of GICs and highlights the clinical rationale for favoring resin-based materials in situations where high strength and durability are required. Similarly, Goldman et al. demonstrated that glass ionomer materials exhibit substantially lower fracture toughness compared with composite resins. Since fracture toughness is a critical property that directly influences a material’s ability to withstand functional stresses and resist crack propagation under masticatory forces, this finding further accentuates the structural deficiencies of GICs.

In the present study, the lowest shear bond strength values were also observed in the GIC group (10.37 ± 4.21 MPa), in full agreement with previous reports [4,18,22,23,24]. These results suggest that GICs possess a limited capacity to establish a durable adhesive interface with monolithic zirconia, and their relatively poor mechanical characteristics may have further contributed to their inferior bonding performance. Taken together, the evidence indicates that conventional GICs are not ideally suited for use as core build-up materials in conjunction with high-strength ceramics such as zirconia, and their clinical application may be more appropriately restricted to temporary or low-stress restorative contexts.

An ideal core build-up material is expected to possess adequate mechanical properties and effectively transmit functional forces. Zirconomer (Zr), developed to overcome the disadvantages of conventional GIC, has been introduced as an alternative material [7]. In a study conducted by Thomas et al. [22], Zirconomer was shown to have higher flexural strength compared with traditional GICs. Similarly, in our study, although the difference between the Zirconomer and GIC groups was not statistically significant, the shear bond strength of Zirconomer (14.19 ± 3.66 MPa) was higher than that of conventional GIC (10.37 ± 4.21 MPa). However, Zirconomer exhibited significantly lower shear bond strength values compared with the NHCR group, and this difference was statistically significant.

The differences in bond strength may be partly explained by the distinct compositions of the materials; the incorporation of zirconia fillers in Zirconomer could account for its improved performance over conventional GIC, while the resin matrix and nano-fillers in NHCR likely contributed to its superior bonding properties [4]. Recent international studies have shown that the incorporation of additives such as chitosan, glass fibers, and graphene oxide into conventional glass ionomer cements (GICs) significantly enhances their mechanical performance compared with unmodified formulations. These findings indicate that bio-reinforcement strategies may overcome the structural limitations of GICs and highlight the critical role of material composition in determining the bonding behavior and durability of restorative systems [23,24].

Prabakaran et al. [3] reported that NHCR exhibits superior mechanical properties compared with Zirconomer when used as core build-up materials. This has been further supported by Aditi et al. [7], who found that composite resins bonded to zirconia had significantly higher strength (7.25 MPa) than Zirconomer (0.74 MPa) (*p* ≤ 0.001). Similarly, Nakade et al. [20] showed that composite resins had higher flexural strength than Zirconomer, and these findings are consistent with the present study. Abraham et al. [25] also compared the shear bond strength (SBS) of GIC, Zirconomer, and Luxacore core build-up materials to zirconia, reporting values of 9.51, 13.94, and 17.48 MPa, respectively. Based on these results, they recommended the combined use of chemical and mechanical surface treatments to improve the bond strength between MZ and Zirconomer.

The failure at the interface between monolithic zirconia (MZ) and core build-up materials is generally classified as adhesive, cohesive, or mixed, depending on the exact location at which the separation occurs during testing. Adhesive failures are characterized by debonding strictly at the interface, cohesive failures occur within the material itself, and mixed failures present features of both types. In a study by Aditi et al. [7], detailed analysis of the failure modes revealed 100% adhesive failures in the Zirconomer group, indicating a weaker interfacial bond between this material and zirconia. In contrast, the composite resin group demonstrated a more balanced distribution of failure types, with 43.8% cohesive, 31.2% mixed, and 25.0% adhesive failures, suggesting improved bonding performance and a stronger internal integrity within the material. Similarly, Giti and Zarkari [8] reported 100% cohesive failures in their investigation, in which composite resin was bonded to zirconia discs, further emphasizing the relatively high internal strength of composite resins when compared with glass ionomer-based materials. These findings collectively highlight how the type of core build-up material can significantly influence the nature of failure at the zirconia interface, underlining the importance of material selection in restorative applications.

In this study, the failure modes observed in the Zirconomer group were 5% adhesive, 30% cohesive, and 65% mixed. In the GIC group, no adhesive failures were recorded; instead, 40% were cohesive and 60% were mixed. In the NHCR group, 15% of the failures were adhesive, 40% were cohesive, and 45% were mixed. The distribution of failure modes provides insight into both the internal strength of the core build-up materials and their bonding capacity with the zirconia surface. The high percentage of mixed (65%) and cohesive (30%) failures in the Zirconomer group suggests that the material formed only a limited bond with zirconia and may also lack sufficient structural strength. Similarly, the complete absence of adhesive failure in the GIC group, combined with a predominance of cohesive and mixed failures, indicates that this material also exhibited poor adhesion to the zirconia surface and had relatively low mechanical properties. In contrast, the more balanced distribution of failure types in the NHCR group (15% adhesive, 40% cohesive, 45% mixed) reflects both a strong internal structure and a more stable bond to zirconia. These findings support the clinical advantage of NHCR as core build-up materials [26].

Since this study focused on monolithic zirconia as the restorative material, its clinical performance is particularly relevant. Beyond the in vitro context, long-term clinical data are essential to validate the performance of zirconia restorations. In this regard, Lolos et al. [27] retrospectively evaluated 1143 zirconium oxide restorations over a 5-year period and reported an overall survival rate of 96.3%, with monolithic restorations achieving a 100% survival rate compared to 95.8% for layered restorations, where failures were mainly related to veneer chipping. These findings support the clinical reliability of monolithic zirconia, further reinforcing its relevance in prosthetic applications and aligning with the high bond strength values observed in our study. Moreover, Beketova et al. [28] demonstrated that the incorporation of zirconia nanoparticles into contemporary dental luting cements enhanced their physicochemical properties and shear bond strength to monolithic zirconia, highlighting how material innovations can further optimize the long-term clinical success of zirconia-based restorations.

This study has several limitations. Firstly, it was conducted under in vitro conditions, which limits the simulation of complex biological and mechanical factors encountered in the clinical setting (e.g., fluctuating moisture, temperature, pH, masticatory forces, and biofilm formation). In addition, thermocycling and artificial aging protocols were intentionally not included, as the primary aim was to establish a baseline comparison of shear bond strength between zirconia and different core build-up materials. Nevertheless, such protocols are essential for mimicking the clinical environment, and future studies should incorporate them to provide more clinically relevant outcomes. In this study, sandblasting was selected as the sole surface treatment together with a single self-adhesive dual-cure resin cement (RubySE Cem), which contains the 10-MDP monomer. This choice was made to ensure methodological standardization and reliable bonding to zirconia. Nevertheless, other surface conditioning methods (e.g., tribochemical silica coating, laser irradiation, plasma treatment) and alternative resin systems with different adhesive compositions or protocols may also influence bonding outcomes and therefore warrant further investigation. Since only one commercial brand was used for each core build-up material, the findings are specific to these products and cannot be generalized to all similar materials. Furthermore, this study evaluated only the shear bond strength; other loading types (e.g., microtensile, flexural) and long-term durability or aging tests were not included. Therefore, to ensure the clinical applicability of these findings, more advanced and long-term clinical studies are needed.

## 5. Conclusions

Within the limitations of this study, the findings emphasize the importance of selecting appropriate core build-up materials to ensure the long-term success of zirconia-based restorations. In particular, nanohybrid composite resins demonstrated superior bonding potential, while zirconia-reinforced glass ionomer cements showed promise as alternatives to conventional GICs. Clinically, these results underline how material choice can directly influence the durability of zirconia restorations. Future research should focus on optimizing the bonding performance of modified glass ionomer systems and validating these outcomes through long-term clinical conditions.

## Figures and Tables

**Table 1:** Composition and manufacturer information of the materials used in this study.

Material	Type	Manufacturer (Country)	Main Composition *
RubyCompNano (NHCR)	Nanohybrid composite resin	Inci Dental, Turkey	Bis-GMA, UDMA, silica/zirconia nano-fillers
Ketac Molar (GIC)	Conventional glass ionomer cement	3M ESPE, Germany	Fluoroaluminosilicate glass, polyacrylic acid, tartaric acid
Zirconomer	Zirconia-reinforced GIC	Shofu, Japan	Fluoroaluminosilicate glass + zirconia fillers, polyacrylic acid, pigments
RubySE Cem	Self-adhesive dual-cure resin cement	Inci Dental, Turkey	10-MDP monomer, resin matrix, inorganic fillers

* The main composition data were obtained from the manufacturers’ product information sheets.

**Table 2:** Descriptive statistics of shear bond strength (MPa) for each core material group.

Group	Mean ± SD (MPa)	Median (MPa)	Min (MPa)	Max (MPa)
Zirconomer	14.19 ± 3.66 ^a^	13.58	7.23	20.48
GIC	10.37 ± 4.21 ^a^	10.22	2.6	17.04
NHCR	48.32 ± 12.49 ^b^	48.76	23.54	71.01

Different superscript letters (^a^, ^b^) indicate statistically significant differences between groups (Dunn’s test, *p* < 0.05). Identical letters indicate no significant difference. SD: Standard deviation; Min: Minimum; Max: Maximum; MPa: Megapascal.

## Data Availability

The datasets used and/or analyzed during the current study are available from the corresponding author on reasonable request.

## References

[1] Landys B., Jonasson P., Kvist T. (2015). Long-term survival of endodontically treated teeth at a public dental specialist clinic. J. Endod..

[2] Warangkulkasemkit S., Pumpaluk P. (2019). Comparison of physical properties of three commercial composite core buildup materials. Dent. Mater..

[3] Prabakaran P., Laxmipriya C.H., Annapoorna B.S. (2022). Comparative evaluation of shear bond strength of various core build-up materials in maxillary anterior teeth—An in vitro study. J. Indian Dent. Res..

[4] Alshabib A., Jurado C.A., Azpiazu-Flores F.X., Aldosary K., Tsujimoto A., Algamaiah H. (2024). Mechanical properties and degree of conversion of resin-based core build-up materials and short fiber-reinforced flowable resin-based composite. Dent. Mater. J..

[5] Marković D., Petronijević B., Blažić L., Šarčev I., Atanacković T. (2011). Bond strength comparison of three core build-up materials used to restore maxillary incisor teeth. Contemp. Mater..

[6] Nujella B.P., Choudary M.T., Reddy S.P., Kumar M.K., Gopal T. (2012). Comparison of shear bond strength of aesthetic restorative materials. Contemp. Clin. Dent..

[7] Aditi P., Mehta S., Raj R. (2023). Comparative evaluation of shear bond strength at the interface of monolithic zirconia with two distinct core build-up materials: An in vitro study. J. Indian Prosthodont. Soc..

[8] Giti R., Zarkari R. (2019). The effect of a zirconia primer on the shear bond strength of Y-TZP ceramic to three different core materials using a self-adhesive resin cement. J. Indian Prosthodont. Soc..

[9] Rüttermann S., Alberts I., Raab W.H.M., Janda R.R. (2011). Physical properties of self-, dual-, and light-cured direct core materials. Clin. Oral Investig..

[10] Kumar L., Pal B., Pujari P. (2015). An assessment of fracture resistance of three composite resin core build-up materials on three prefabricated non-metallic posts cemented in endodontically treated teeth: An in vitro study. PeerJ.

[11] Gu Y.W., Yap A.U., Cheang P., Khor K.A. (2005). Effects of incorporation of HA/ZrO_2_ into glass ionomer cement (GIC). Biomaterials.

[12] Dheeraj M., Johar S., Jandial T., Sahi H., Verma S. (2019). Comparative evaluation of compressive strength and diametral tensile strength of zirconomer with GIC and amalgam. J. Adv. Med. Dent. Sci. Res..

[13] Tavakolizadeh S., Dehghan M., Ghoveizi R., Fayyazi A. (2021). Shear bond strength of zirconia ceramic to four different core materials—An in vitro study. J. Dent..

[14] Vidotti H.A., Pereira J.R., Insaurralde E., Placa L.F., Delben J.R., do Valle A.L. (2017). Influence of thermal and mechanical fatigue on the shear bond strength of different all-ceramic systems. Clin. Exp. Dent. Res..

[15] Valandro L.F., Özcan M., Bottino M.C., Bottino M.A., Scotti R., Della Bona A. (2006). Bond strength of a resin cement to high-alumina and zirconia-reinforced ceramics: The effect of surface conditioning. J. Adhes. Dent..

[16] Wolfart M., Lehmann F., Wolfart S., Kern M. (2007). Durability of the resin bond strength to zirconia ceramic after using different surface conditioning methods. Dent. Mater..

[17] Bonilla E.D., Mardirossian G., Caputo A.A. (2000). Fracture toughness of various core build-up materials. J. Prosthet. Dent..

[18] Capp N.J., Warren K. (1992). An advantage of the direct post and core technique. J. Prosthet. Dent..

[19] Mitra S.B., Wu D., Holmes B.N. (2003). An application of nanotechnology in advanced materials. J. Am. Dent. Assoc..

[20] Nakade P., Thaore S., Bangar B., Grover I., Alharethi N., Adsure G., Kulkarni D. (2024). Comparative evaluation of fracture toughness and flexural strength of four different core build-up materials: An in vitro study. J. Contemp. Dent. Pract..

[21] Agrawal A., Mala K. (2014). An in vitro comparative evaluation of physical properties of four different types of core materials. J. Conserv. Dent..

[22] Thomas H.A., Singh N., Thomas A.M., Masih S., Cherian J.M., Varghese K.G. (2024). Effect of protective coating agents on microleakage and flexural strength of glass ionomer cement and zirconomer—An in vitro study. Eur. Arch. Paediatr. Dent..

[23] Thenumkal E., Sahoo N., Chopra S., Joshi P., Mustafa M., Kumari D. (2025). Evaluation of the Mechanical Properties and Fluoride Release Profiles of a GIC and Chitosan-Modified GIC. J. Contemp. Dent. Pract..

[24] Sari F., Ugurlu M. (2023). Reinforcement of resin-modified glass-ionomer cement with glass fiber and graphene oxide. J. Mech. Behav. Biomed. Mater..

[25] Abraham R., Annapoorni H., Lakshmanan G., Karthik L. (2019). Comparative evaluation of shear bond strength of different core build-up materials with zirconia using a self-adhesive resin cement: An in vitro study. Int. J. Med. Health Res..

[26] Musa D.B., Ereifej N.S. (2023). The influence of core build-up materials on biaxial flexural strength of monolithic strength-gradient zirconia: An in vitro study. BMC Oral Health.

[27] Lolos D., Mihali S.G., Dinu S., Mitariu M., Tudor A., Oancea R. (2025). Retrospective long-term survival rate and clinical performance of zirconium oxide restorations over the past 5 years: A comparative study between single crowns and fixed dental prostheses. Medicina.

[28] Beketova A., Tzanakakis E.-G.C., Vouvoudi E., Anastasiadis K., Rigos A.E., Pandoleon P., Bikiaris D., Tzoutzas I.G., Kontonasaki E. (2023). Zirconia Nanoparticles as Reinforcing Agents for Contemporary Dental Luting Cements: Physicochemical Properties and Shear Bond Strength to Monolithic Zirconia. Int. J. Mol. Sci..

